# Thirty years of the health care service for ostomy patients in Juiz de Fora and surroundings

**DOI:** 10.1590/0100-6991e-20202644

**Published:** 2021-01-13

**Authors:** MAURO TOLEDO SIRIMARCO, BRENO HENRIQUE XAVIER DE MORAES, DENISE RABELLO LOVISI SALES DE OLIVEIRA, ALFEU GOMES DE OLIVEIRA, PATRICIA APARECIDA FONSECA SCHLINZ

**Affiliations:** 1 - Universidade Federal de Juiz de Fora, Faculdade de Medicina - Departamento de Cirurgia - Juiz de Fora - MG - Brasil; 2 - Universidade Federal de Juiz de Fora, Faculdade de Medicina - Juiz de Fora - MG - Brasil; 3 - Prefeitura Municipal de Juiz de Fora, Secretaria de Saúde - Departamento de Clínicas Especializadas - Juiz de Fora - MG - Brasil

**Keywords:** Ostomy, Postoperative Complications, Colorectal Surgery, General Surgery, Urology, Estomia, Complicações Pós-Operatórias, Cirurgia Colorretal, Cirurgia Geral, Urologia

## Abstract

**Objective:**

to establish the epidemiological profile of ostomized patients treated at the Health Care Service for Ostomy Patients in Juiz de Fora and region (SASPO/JF) and to quantify the pathologies that led to the stoma as well as the ostomy-related complications.

**Method:**

a retrospective study was carried out with the analysis of 496 medical records of patients registered at HCSOP/JF over 30 years and who remained in at the service in June 2018. The following variables were considered: age, sex, pathology that led to the stoma, type, time, location and complications of stomas.

**Results:**

53.43% were male patients and 46.57% female. The average age was 56.24 years among men and 58.40 years among women. Eight patients had two types of ostomies simultaneously and a total of 504 ostomies were as follows: 340 colostomies (67.46%), 117 ileostomies (23.21%) and 47 urostomies (9.33%). Additionally, 47.65% of the colostomies and 76.92% of the ileostomies were temporary, while all urostomies were permanent. In 70.24% of cases, the reason for making the stoma was malignancy. There were 277 stomas with one or more complications (54.96%)*.*

**Conclusions:**

most of the ostomized patients were over 50 years old and the main diagnosis that led to the stoma was malignancy. Ileostomies had a higher percentage of complications than colostomies and urostomies and, for all types of stomas, the most frequent complication was dermatitis.

## RESUMO

**Objetivo:**

elaborar o perfil epidemiológico dos pacientes estomizados atendidos no Serviço de Atenção à Saúde da Pessoa Ostomizada de Juiz de Fora e região (SASPO/JF) e quantificar tanto as patologias que levaram à confecção, quanto as complicações presentes nas estomias.

**Método:**

realizado estudo retrospectivo com análise de 496 prontuários de pacientes cadastrados no SASPO/JF ao longo de 30 anos e que permaneciam em atendimento no serviço em junho de 2018. Foram consideradas as seguintes variáveis: idade, sexo, patologia que levou à confecção do estoma, tipo, caráter temporal, localização e complicações das estomias.

**Resultados:**

53,43% dos pacientes eram do sexo masculino e 46,57% do sexo feminino. A média de idade entre os homens foi de 56,24 anos e entre as mulheres foi de 58,40 anos. Oito pacientes apresentaram dois tipos de estomias simultaneamente e o total de 504 estomias foi distribuído da seguinte forma: 340 colostomias (67,46%), 117 ileostomias (23,21%) e 47 urostomias (9,33%). Além disso, 47,65% das colostomias e 76,92% das ileostomias foram temporárias, enquanto todas as urostomias foram permanentes. Em 70,24% dos casos, o motivo para confecção do estoma foi a neoplasia maligna. Foram encontrados 277 estomas com uma ou mais complicações (54,96%).

**Conclusão:**

as estomias predominaram em pacientes com mais de 50 anos e o principal diagnóstico que levou à confecção dos estomas foi a neoplasia maligna. As ileostomias apresentaram maior percentual de complicações do que as colostomias e urostomias e, para todos os tipos de estomas, a complicação mais frequente foi a dermatite.

## INTRODUCTION

In June 1988, in the city of Juiz de Fora, in the Brazilian State of Minas Gerais, the Ostomy Patient Care Program (PAPO) was implemented, covering populations in the cities of Juiz de Fora, Ubá, Barbacena, and Leopoldina. The program’s activities began with the gathering of an interprofessional team composed of one doctor from the University Hospital of the Federal University of Juiz de Fora, one nurse from the Municipal Health Secretariat (SMS), one social worker from the National Institute of Medical Assistance for Social Security (INAMPS), and one psychologist from the National Institute of Social Security (INSS). The program was created at the initiative of the professionals themselves^1^. With the creation of the Brazilian Unified Health System (SUS) by the Federal Constitution of 1988, the right of the ostomy patient to health care become a duty of the State, and must be carried out from the preoperative period until the individual’s reintegration into society^2^. 

As of 1993, the program had its name changed to Health Care Service for Ostomy Patients (SASPO/JF), taking place at the Department of Specialized Clinics of the Municipality of Juiz de Fora, linked to the Unified Health System (SUS). The service assists patients with elimination urinary (urostomies) and intestinal (ileostomies and colostomies) stomas who reside in the urban and rural areas of the 38 cities in the Juiz de Fora macro-region. 

The legal basis for the continuity of the activities of this service was Ordinance 116/1993 of the Ministry of Health, which brought the expanded concept of health and ensured the granting of devices (collection bags) to patients with ostomy by the Unified Health System (SUS)^1^ ^,^ ^3^. 

Ordinance number 400/2009 of the Ministry of Health established the “National Guidelines for the Attention to the Health of Ostomy People within the scope of the Unified Health System - SUS”. According to this ordinance, SASPO/JF is part of the Ostomy Care Service II, providing specialized and interdisciplinary assistance, with the objective of rehabilitating the patient, including guidance for self-care, prevention, treatment of ostomy complications, training, and supply of collection, protection, and safety equipment. The service must have at least the following human resources: doctor, nurse (with training in assistance to people with stomas), social worker, psychologist, and nutritionist. The number of professionals must be appropriate to the demands and the territorial area covered by the service, giving priority to the highest proportion of nurses in the team. Professionals do not need to be exclusive to the service^1^ ^,^ ^4^. 

The services provided by SASPO/JF consist of preoperative and postoperative consultations, medical, psychosocial, nutritional, and nursing monitoring, in addition to the distribution of devices. In the preoperative consultation, there is guidance on functioning, biopsychosocial assessment, and demarcation of the abdomen for making the stoma. In postoperative care, initiated while the patient is still in the hospital or in a home visit, the patient and his family are instructed in the necessary documentation for registration, follow-up in the service, and correct use of the devices. In addition to nursing assistance in the periodic stoma evaluation, there is assistance from a social worker, a psychologist, and a nutritionist. There are also support meetings for patients and their families. Through lectures, therapeutic, psychopedagogical, and educational groups addressing themes inherent to the condition of the ostomy patient, there is a stimulus to self-care, to the patient’s adaptation to the new condition, to reintegration into society, and to social life^1^. 

In 2018, the SASPO/JF completed its thirtieth anniversary. Since the foundation, all services have been documented in medical records, resulting in a rich collection for historical analysis. The present study aims to evaluate the epidemiological profile of ostomized patients seen at the service and to quantify both the diseases that led to the preparation and the complications associated with ostomies.

## METHODS

From June 1988 to June 2018, the SASPO/JF, located in the SUS Specialized Clinics Department in the city of Juiz de Fora (MG), assisted 2,805 people with stomas. During this period, the stoma was reverted in 911 patients, 92 left the service, 24 ostomized patients were transferred, and 1,251 patients died. We analyzed 527 medical records of patients registered over 30 years and who remained in the service in June 2018, of which we excluded 31 medical records with illegible notes or with incomplete information according to the objectives of the study. We filled out previously prepared forms, and analyzed data from 496 medical records. We considered the variables age, sex, disease that led to the construction of the stoma, type, temporal character, location, and complications of the stomas.

## RESULTS

**Figure 1:**
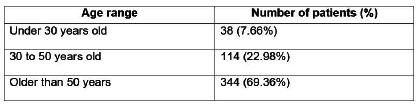
Distribution of 496 patients according to age range.

 Of the 496 medical records analyzed, 265 were from male patients (53.4%) and 231 from females (46.6%). The average age among men was 56.2 years, while among women it was 58.4 years. The general average age was 57.2 years and there was a predominance of patients over 50 years old, as shown in Figure 1.

Eight patients had two simultaneous types of ostomies, comprising a total of 504 ostomies, distributed in 340 colostomies (67.5%), 117 ileostomies (23.2%), and 47 urostomies (9.3%). Of the patients with simultaneous stomas, two had ileostomy and colostomy, two others had ileostomy and urostomy, and four had both colostomy and urostomy.

**Figure 2:**
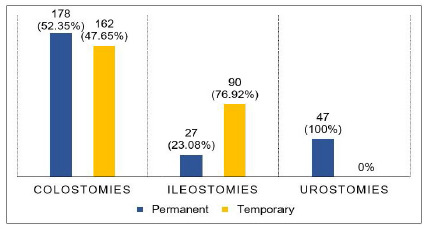
Classification of stomas according to temporal character.

**Figure 3:**
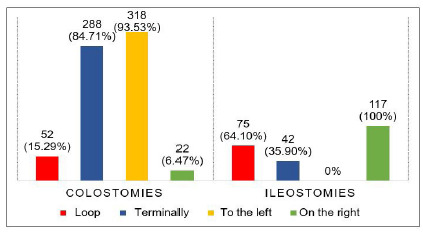
Types and location of intestinal stomas.

Among the 340 colostomies, there was a predominance of stomas made terminally and to the left, the majority being permanent. Of the 117 ileostomies, most were made in loop and on the right, with a predominance of temporary stomas. All urostomies were permanent (Figures 2 and 3).

**Figure 4:**
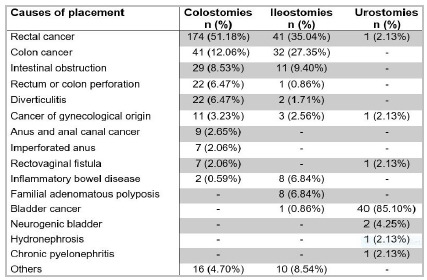
Causes that led to the placement of 340 colostomies, 117 ileostomies and 47 urostomies.

The most frequent cause that led to the placement of 340 colostomies was malignancy, occurring in 235 cases (69.1%), of which rectal cancer was the most reported, followed by colon cancer. Cancer of gynecological origin and cancer of the anus/anal canal occurred in smaller percentages. Less frequent causes were observed, such as intestinal obstruction, rectum or colon perforation (due to foreign body or trauma), diverticulitis, imperforated anus, rectovaginal fistula, and inflammatory bowel disease, the occurrence rates of which can be seen in Figure 4. We also identified other causes that led to the colostomy creation: peritonitis (4 cases), megacolon (2), Fournier’s syndrome (2), acute abdomen (2), Hirschsprung’s disease, stenosis of perineal colostomy, laceration of the rectum, polyp of rectum, rectal polyposis, and proctitis.

As for the 117 ileostomies, the prevailing cause was also malignancy, with 77 cases (65.8%), rectal and colon cancer being the most frequent, while bladder cancer and gynecological cancer displayed lower percentages. We also observed less frequent causes, such as intestinal obstruction, familial adenomatous polyposis, inflammatory bowel disease, diverticulitis, and colon perforation, as shown in Figure 4. Other causes that led to the creation of ileostomy were fistula (3), acute appendicitis (2), intestinal necrosis, colon necrosis, ischemic colitis, anal and rectal bleeding, and chronic pancreatitis.

Among the 47 urinary stomas, malignant neoplasm was responsible for 42 urostomies (89.4%), bladder cancer being the most frequent. Less frequent causes observed were neurogenic bladder, rectal cancer, gynecological cancer, rectovaginal fistula, hydronephrosis (Warkany’s syndrome), and chronic pyelonephritis (Figure 4). 

In 504 stomas (colostomies, ileostomies, and urostomies), we found no complications in 227 (45.0%), one complication in 171 (33.9%), and two or more complications in 106 (21.0%), revealing a total of 277 stomas with complications (54.9%).

**Figure 5:**
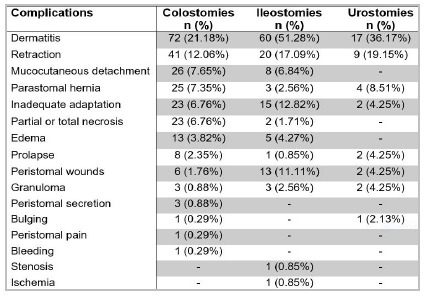
Frequency of complications in 340 colostomies, 117 ileostomies e 47 urostomies.

We observed one or more complications in 179 (52.6%) of the 340 colostomies and in 73 (62.4%) of the 117 ileostomies. Thus, of the 457 intestinal ostomies, 252 (55.1%) had complications, while 25 (53.2%) of the 47 urostomies had at least one complication. Dermatitis was the most prevalent complication among intestinal and urological derivations (Figure 5).

## DISCUSSION

The International Ostomy Association (IOA) projects one person with an ostomy for every 1,000 inhabitants in countries with a good level of health care, which may be much lower in less developed countries. In Brazil, there were more than 207,000 estimated people with such derivations in 2018. It is emphasized that this estimate was calculated considering elimination ostomies^5^. 

Among all colostomized, ileostomized, and urostomized patients evaluated in this study, there was a predominance of males, 6.86% more than females. In the literature, there are authors who describe male predominance^6^ ^-^ ^11^, while others observe a preponderance of females^12^ ^-^ ^15^. Our study demonstrated that malignant neoplasm was the main cause of creation of the derivations, and dermatitis, the most frequent complication in the three types of stoma. According to the World Health Organization (WHO)^16^, with the progression of the population’s life expectancy, the number of new cancer cases continues to increase, since aging is one of the main factors in the process of oncogenesis. This is reflected in the average age and higher age groups of patients with intestinal and urological derivations, as found in this and in other studies^6^ ^-^ ^15^ ^,^ ^17^.

The main cause leading to intestinal derivations (colostomies and ileostomies) was colorectal cancer. The proportion found is consistent with the literature in several studies, as a consequence of the high incidence of intestinal cancer^6,8 13,15,18,19,20^. Without considering non-melanoma skin tumors, in Brazil, colon and rectal cancer is the second most frequent in both males and females, behind prostate and breast cancers, respectively. For each year of the 2020-2022 triennium, 20,520 new cases of colon and rectal cancer are estimated in men, and 20,470, in women^21^. Bray et al. and Ferlay et al., assessing the incidence of 36 types of cancer in 185 different countries, estimate that one million men were affected by colon and rectal cancer in 2018, while 800,000 new cases occurred among women^22^ ^,^ ^23^. 

Most cases of colorectal cancer are related to the patient’s lifestyle, such as diet, smoking, physical inactivity, and obesity^21^ ^,^ ^24^ ^,^ ^25^. However, there are factors of hereditary and genetic origin, in addition to occupational exposure, which increase the risk of developing this cancer^21^. Age equal to or greater than 50 years is an additional risk, since there is greater exposure to risk factors over the years; the number of stomas is thus higher in this population^10^ ^,^ ^16^ ^,^ ^24^ ^,^ ^25^. The American Cancer Society and the Brazilian National Cancer Institute (INCA) establish that advanced age is one of the main risk factors for the development of colon and rectal cancer, in addition to other conditions such as familial adenomatous polyposis and inflammatory bowel disease^21^ ^,^ ^25^. 

The results of this research reveal that the clinical profile of ostomized patients corroborates the findings obtained in other studies related to the theme, which have shown a predominance of malignant neoplasms, mainly colorectal cancer, as the main medical diagnosis requiring surgical intervention and creation of an intestinal elimination stoma. The Brazilian Association of Ostomy Patients (ABRASO), the International Ostomy Association (IOA), and the United Ostomy Association (UOA) point out that oncological causes predominate in the stoma indication; however, inflammatory, traumatic, and congenital causes are also important^15^. In addition, intestinal derivation is indicated in cases of intestinal obstruction, colon perforations, traumas, fistulas, anastomoses protection, and congenital malformations^26^ ^,^ ^27^ ^,^ ^28^. We identified seven cases of imperforate anus, which explains the creation of colostomy in patients of both sexes in the first days of life. 

Several studies indicate that colostomy is the most common type of elimination stoma, followed by ileostomies, as observed in this study^6^ ^-^ ^8^ ^,^ ^10^ ^,^ ^11^ ^,^ ^13^ ^-^ ^15^ ^,^ ^17^ ^,^ ^20^ ^,^ ^25^ ^,^ ^26^. Most colostomies were terminally and permanently made, predominantly on the left, as found by other authors^5^ ^,^ ^10^ ^,^ ^12^ ^,^ ^17^ ^,^ ^19^ ^,^ ^20^ ^,^ ^29^. Terminal colostomy is often indicated when a long-term stoma is predicted, as in advanced rectal cancer or peritonitis^13^. lleostomies, on the other hand, were mostly created in loop and temporarily, predominantly to the right, being consistent with the literature^13^ ^,^ ^19^ ^,^ ^20^ ^,^ ^29^. 

Of 457 intestinal stomas, 252 had one or more complications (55.1%) and this is comparable with other works, who report complication rates ranging between 23.0% and 60.0%^13^ ^,^ ^17^ ^,^ ^26^ ^,^ ^30^. The literature reveals a higher percentage of complications in ileostomies compared with colostomies^6^ ^,^ ^14^ ^,^ ^26^, consistent with the findings of this study: ileostomies with 62.4% and colostomies with 52.6% of complications. This is explained by the liquid consistency and greater volume of the ileal content in relation to the colonic content, easily drained through the stoma and affecting the patient’s skin. This alkaline liquid causes serious wounds, hampering correct adaptation of the bag to the abdominal wall, further favoring the contact of the contents with the skin^27^ ^,^ ^30^. An evidence of this was the greater occurrence of peristomal wounds in ileostomies (11.1%) when compared with colostomies (1.7%), as well as the occurrence of dermatitis. 

Peristomal dermatitis was the most common complication in intestinal derivations (51.2% in ileostomies and 21.1% in colostomies), as demonstrated by other authors. The high rate of dermatitis has been correlated with a higher frequency of exchange of the collection equipment and also with its inadequate handling, which may be a consequence of the long ostomy period of the monitored patients^6^ ^,^ ^7^ ^,^ ^13^ ^,^ ^20^ ^,^ ^26^. The second most frequent complication was retraction, with a rate of 17.1% in ileostomies and 12.0% in colostomies, percentages similar to other surveys^6^ ^,^ ^26^ ^,^ ^30^. There are no uniform criteria in the literature to assess the causes of retraction. However, thickened mesentery makes it difficult to mobilize the intestinal loop, and a greater panicle in obese patients exerts traction on the intestinal wall, which can lead to retraction^17^. As this is a documentary research, we could not assess whether there was any technical difficulty in creating the stoma, since there was no such description in the medical records. 

Prolapse in an uncommon complication of stomas and may be associated with parastomal hernia^19^. In the intestinal ostomies analyzed, prolapse reached a percentage of 2.3% in colostomies and 0.8% in ileostomies, while parastomal hernia was present in 7.3% of colostomies and in 2.5% of ileostomies. In other studies, there was also a higher occurrence of these complications in colonic derivations than in ileal ones^6^ ^,^ ^13^ ^,^ ^14^ ^,^ ^17^ ^,^ ^18^ ^,^ ^26^ ^,^ ^30^. It is important to monitor ostomized patients for the appearance of bulging, parastomal hernias, and prolapses, which cause discomfort, pain, and even poor adaptation to the bag.

Inadequate adaptation occurred in both intestinal and urological derivations, located in cutaneous depressions, close to the surgical or umbilical scar, to the iliac crest, among other anatomical accidents, allowing the leakage of the content drained by the ostomy. The inadequate choice of the stoma’s site is more common in urgent surgeries, emphasizing the importance of preoperative planning for a proper location, which is vital for the patient’s quality of life^6^ ^,^ ^14^ ^,^ ^27^ ^,^ ^31^. 

Urostomies are usually held when there is cancer of the bladder or urethra, congenital bladder abnormalities, or spine alterations, such as spina bifida or neural tube defects^32^. This research showed that most patients with urinary diversion had bladder cancer. In 2018, according to Bray et al. and Ferlay et al., bladder cancer was the sixth most common in males and the seventeenth in females^22^ ^,^ ^23^. The number of new cases of bladder cancer estimated for Brazil, for each year of the 2020-2022 triennium, is 7,590 cases in men and 3,050 in women. These values correspond to an estimated risk of 7.23 new cases per 100,000 men and 2.80 per 100,000 women, which may lead to an increase in the number of urostomized patients^21^. According to the American Cancer Society^25^, 90.0% of cases of this cancer require surgery, either alone or in combination with other therapies, such as immunotherapy or chemotherapy, and in some cases a urostomy may be necessary^29^. Resection of the bladder can be partial or total, and more advanced tumors may require complete removal of this organ, termed radical cystectomy^25^ ^,^ ^33^. The WHO recommends that urologists consider definitive urinary diversion for patients who have undergone radical cystectomy for invasive bladder cancer^34^. In this perspective, this study showed that all urostomized patients had permanent derivations. The overall complication rate in urinary diversions was 53.1%, while in the literature the rates vary from 20.0% to 65.0%^20^ ^,^ ^31^ ^,^ ^32^ ^,^ ^35^ ^,^ ^36^. In line with other analyzes, dermatitis was the most common complication found in urostomies, in 36,1% of cases. As with intestinal ostomies, the presence of dermatitis may be a consequence of the high frequency of exchange of the collection equipment and its inadequate handling^20^ ^,^ ^31^ ^,^ ^32^. In the literature, it is rare to find the occurrence of prolapse and the presence of granuloma, which we observed in 4.2% in urinary stomas, complications that may be related to the length of time the stomas remained^6^ ^,^ ^31^ ^,^ ^32^. 

In view of the high prevalence of debilitating diseases and the fragility that an ostomy can cause to the patient, the interdisciplinary service created to care for ostomized patients in the Juiz de Fora region has been providing relevant services to patients with colostomies, ileostomies, and urostomies for over thirty years, aiming at better quality of life for the patients.

## References

[1] Oliveira AL (2017). Qualidade de Vida Relacionada à Saúde e Perfil Nutricional de Portadores de Derivação Intestinal: colostomia e ileostomia.

[2] Santos VLCG (2006). Cuidando do estomizado: análise da trajetória no ensino, pesquisa e extensão.

[3] Brasil. Ministério da Saúde (1993). Portaria no 116, de 9 de setembro de 1993. Inclui no Sistema de Informações Ambulatoriais do Sistema Único de Saúde-SAI-SUS a concessão dos equipamentos de órteses, próteses e bolsas de colostomia constantes do anexo único.

[4] Brasil. Ministério da Saúde (2009). Portaria no 400, de 16 de novembro de 2009. Estabelece diretrizes nacionais para a atenção à saúde das pessoas ostomizadas no âmbito do Sistema Único de Saúde - SUS, a serem observadas em todas as unidades federadas, respeitadas as competências das três esferas de gestão.

[5] Brasil. Ministério da Saúde (2019). Guia de Atenção à Saúde da Pessoa com Estomia. Brasília: Secretaria de Atenção Especializada em Saúde; Departamento de Atenção Especializada e Temática; Coordenação-Geral de Saúde da Pessoa com Deficiência.

[6] Santos CHM, Bezerra MM, Bezerra FMM, Paraguassú BR (2007). Perfil do Paciente Ostomizado e Complicações Relacionadas ao Estoma. Rev bras Coloproct.

[7] Silva CRDT, Andrade EMLR, Luz MHBA, Andrade JX, Silva GRF (2017). Qualidade de vida de pessoas com estomias intestinais de eliminação. Acta Paul Enferm.

[8] Schwalm MT, Ceretta LB, Farias BM, Bonfanti MDP, Zimmermann KCG, Perfoli R (2013). Perfil das pessoas ostomizadas atendidas na clínica escola de enfermagem da Universidade do Extremo Sul Catarinense - UNESC. Rev Iniciação Científica.

[9] Rubio-Perez I, Leon M, Pastor D, Diaz Dominguez J, Cantero R (2014). Increased postoperative complications after protective ileostomy closure delay An institutional study. World J Gastrointest Surg.

[10] Moraes JT, Assunção RS, Sá FS, Lessa ER, Corrêa LS (2016). Perfil de pessoas estomizadas de uma região de saúde mineira. Enferm Foco.

[11] Oliveira AL, Mendes LL, P M, Leite ICG (2017). Cross-cultural Adaptation and Validation of the Stoma Quality of Life Questionnaire for Patients With a Colostomy or Ileostomy in Brazil A Cross-sectional Study. Ostomy Wound Manage.

[12] Stumm EMF, Oliveira ERA, Kirschner RM (2008). Perfil de pacientes ostomizados. Scientia Medica.

[13] Caricato M, Ausania F, Ripetti V, Bartolozzi F, Campoli G, Coppola R (2007). Retrospective analysis of long-term defunctioning stoma complications after colorectal surgery. Colorectal Dis.

[14] Harilingam M, Sebastian J, Twum-Barima C, Boshnaq M, Mangam S, Khushal A (2017). Patient-related factors influence the risk of developing intestinal stoma complications in early post-operative period. ANZ J Surg.

[15] Freitas Nascimento MV, Oliveira da Vera S, Rodrigues Silva MC, Ferreira de Morais F, Rangel Andrade EML, Nogueira Bastos SNMA (2018). Perfil sociodemográfico e clínico de pacientes em pósoperatório de confecção de estomas intestinais de eliminação. Ciencia y Enfermeira.

[16] World Health Organization ? (2020). WHO report on cancer: setting priorities, investing wisely and providing care for all.

[17] Arumugam PJ, Bevan L, Macdonald L, Watkins AJ, Morgan AR, Beynon J (2003). A prospective audit of stomas-analysis of risk factors and complications and their management. Colorectal Dis.

[18] Formijne Jonkers HA, Draaisma WA, Roskott AM, van Overbeeke AJ, Broeders IA, Consten EC (2012). Early complications after stoma formation a prospective cohort study in 100 patients with 1-year follow-up. Int J Colorectal Dis.

[19] Cruz GMG, Constantino JRM, Chamone BC, Andrade MMA, Gomes DMBM (2008). Complicações dos Estomas em Câncer Colorretal Revisão de 21 Complicações em 276 Estomas Realizados em 870 Pacientes Portadores de Câncer Colorretal. Rev bras Coloproct.

[20] Dantas FG, Souza AJG, Melo GSM, Freitas LS, Lucena SKP, Costa IKF (2019). Prevalência de complicações em pessoas com estomias urinárias e intestinais. Revista Enfermagem Atual.

[21] Instituto Nacional de Câncer José Alencar Gomes da Silva (2019). Estimativa 2020: incidência de câncer no Brasil.

[22] Bray F, Ferlay J, Soerjomataram I, Siegel RL, Torre LA, Jemal A (2018). Global cancer statistics 2018: GLOBOCAN estimates of incidence and mortality worldwide for 36 cancers in 185 countries. CA Cancer J Clin.

[23] Ferlay J, Colombet M, Soerjomataram I, Mathers C, Parkin DM, Pineros M (2019). Estimating the global cancer incidence and mortality in 2018: GLOBOCAN sources and methods. Int J Cancer.

[24] Guimarães RM, Muzi CD, Boccolini CS, Boccolini PMM, Boeira SF (2012). Tendência da mortalidade por câncer de cólon e reto no Brasil segundo sexo, 1980-2009. Cad Saúde Colet.

[25] American Cancer Society (2019). Cancer Facts & Figures 2019.

[26] Park JJ, Del Pino A, Orsay CP, Nelson RL, Pearl RK, Cintron JR (1999). Stoma complications The Cook County Hospital experience. Dis Colon Rectum.

[27] Rocha J (2011). Estomias intestinais - (ileostomias e colostomias) e anastomoses intestinais. Medicina (Ribeirão Preto).

[28] Santos OJ, S EN, B AKD, Desterro VS, Silva MVT, Prado RPS (2016). Children and adolescents ostomized in a reference hospital Epidemiological profile. J Coloproctol.

[29] Burch J (2013). Stoma complications an overview. Br J Community Nurs.

[30] Robertson I, Leung E, Hughes D, Spiers M, Donnelly L, Mackenzie I (2005). Prospective analysis of stoma-related complications. Colorectal Dis.

[31] Nordström GM, Borglund E, Nyman CR (1990). Local status of the urinary stoma--the relation to peristomal skin complications. Scand J Urol Nephrol.

[32] Nazarko L (2008). Caring for a patient with a urostomy in a community setting. Br J Community Nurs.

[33] Kataja VV, Pavlidis N, ESMO Guidelines Task Force (2005). ESMO Minimum Clinical Recommendations for diagnosis, treatment and follow-up of invasive bladder cancer. Ann Oncol.

[34] World Health Organization Consensus Conference on Bladder Cancer (2007). Hautmann RE, Abol-Enein H, Hafez K, Haro I, Mansson W, Mills RD, et al Urinary diversion. Urology.

[35] Singh G, Wilkinson JM, Thomas DG (1997). Supravesical diversion for incontinence a long-term follow-up. Br J Urol.

[36] Madersbacher S, Schmidt J, Eberle JM, Thoeny HC, Burkhard F, Hochreiter W (2003). Long-term outcome of ileal conduit diversion. J Urol.

